# Keeping the photon in the dark: Enabling quantum dot dark state control by chirped pulses and magnetic fields

**DOI:** 10.1126/sciadv.adu4261

**Published:** 2025-07-09

**Authors:** Florian Kappe, René Schwarz, Yusuf Karli, Thomas Bracht, Vollrath M. Axt, Saimon F. Covre da Silva, Armando Rastelli, Vikas Remesh, Doris E. Reiter, Gregor Weihs

**Affiliations:** ^1^ Institut für Experimentalphysik, Universität Innsbruck, 6020 Innsbruck, Austria.; ^2^ Cavendish Laboratory, University of Cambridge, Cambridge CB30HE, UK.; ^3^ Condensed Matter Theory, Department of Physics, TU Dortmund, 44221 Dortmund, Germany.; ^4^ Theoretische Physik III, Universität Bayreuth, 95440 Bayreuth, Germany.; ^5^ Institute of Semiconductor and Solid State Physics, Johannes Kepler University Linz, 4040 Linz, Austria.; ^6^ Universidade Estadual de Campinas, Instituto de Física Gleb Wataghin, 13083-859 Campinas, Brazil.

## Abstract

Dark excitons in quantum dots are not directly optically accessible, which has limited their use in practical applications. Nevertheless, they offer promising features such as substantially longer lifetimes compared to bright excitons, making them attractive candidates for quantum information processing. While previous theoretical and experimental studies have explored their potential, their full capabilities remain largely untapped. In this work, we demonstrate an all-optical storage and retrieval of the spin-forbidden dark exciton in a quantum dot from the ground state using chirped pulses and an in-plane magnetic field. Our experimental findings are in excellent agreement with theoretical predictions of the dynamics calculated using state-of-the-art product tensor methods. Our scheme enables an all-optical control of dark states without relying on any preceding decays. This opens up an unexplored dimension for optimal quantum control and time-bin entangled photon pair generation from quantum dots.

## Teaser

Chirped laser pulses and magnetic fields enable all-optical control over dark exciton states in semiconductor quantum dots.

## INTRODUCTION

Semiconductor quantum dots are one of the most promising source of quantum light to establish a quantum network (*1*). Their ability to generate high-quality states of quantum light, such as single photons (*2*, *3*), entangled photon pairs (*4*–*6*), or correlated multiphoton states (*7*), offers immense flexibility in establishing the key functional elements of such a quantum network (*8*). Single photons and entangled photon pairs are also essential resources of optical quantum computing (*9*–*11*), secure communication via quantum key distribution (QKD) (*12*, *13*), and the distribution of quantum information in general.

The solid-state nature of quantum dots allows engineering the spectral properties via the growth process (*14*) or post-fabrication tuning methods (*15*–*18*). In addition to these, the interaction with lattice vibrations (phonons) (*19*–*22*) delivers a challenging but rewarding landscape of quantum states, which even allows for off-resonant state preparation via phonon-mediated processes.

Well-developed excitation protocols (*23*–*29*) and engineered photonic cavity structures (*30*) have put quantum dots at the forefront of quantum emitters producing single photons with excellent properties such as the single-photon purity (*31*, *32*), indistinguishability (*33*), high photon rate (*34*), and control over coherence properties (*35*, *36*).

The photon generation from quantum dots relies on the recombination of bright excitons, i.e., the radiative recombination of an electron/heavy hole pair of opposite spin. On the other hand, quantum dots also host excitons of parallelly oriented spins that are optically inactive, hence called dark excitons (*37*–*41*).

Due to suppression of emission, the dark exciton states exhibit a substantially reduced decay rate, resulting in lifetimes that can be orders of magnitude longer than their optically bright counterparts (*42*, *43*). This attribute makes them ideal candidates for storing and distributing quantum coherence over time in the generation of time-bin entanglement (*44*, *45*) as proposed in (*36*).

Therefore, to exploit the full potential of the dark exciton states, one has to address the challenge of optically accessing and coherently manipulating it. For this, one possibility is to prepare states in higher excited manifolds (*46*, *47*), relying on subsequent decays. However, preparing the dark exciton coherently within elementary, s-shell–like excitations has remained a theoretical proposal (*48*, *49*).

In this work, we discuss and implement a simple and flexible method to prepare and manipulate optically dark states in a quantum dot using chirped picosecond laser pulses and an external in-plane magnetic field (Voigt configuration). We use state-of-the-art simulations based on product tensor methods to obtain deeper insights on the preparation of the dark state in a numerically exact way (*50*, *51*). Our method unlocks control over these often overlooked states, extending the utility of quantum dots as a platform for quantum applications.

## RESULTS

### Characterization of the dark exciton

**Fig. 1.**
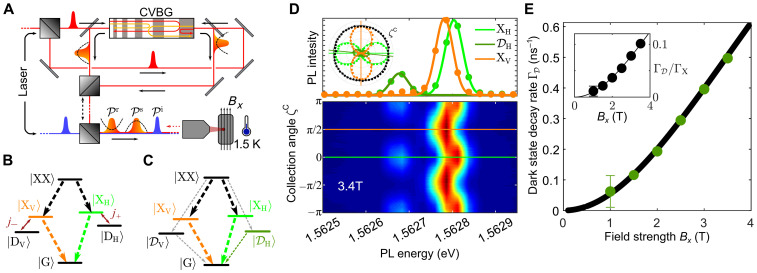
Dark state characterization. (**A**) Sketch of the system and experimental setup: Chirped laser pulses are prepared via a chirped volume Bragg grating (CVBG) and the help of 4f pulse shapers (not shown). Sequences up to three pulses called initialization ( $P^{i}$ , shown in blue), storage ( $P^{s}$ ), and retrieval ( $P^{r}$ ) pulse are sent onto a quantum dot hosted in a cryostat at 1.5 K equipped with a vector magnet. (**B**) Level scheme of the quantum dot within the bare-state basis, consisting of the ground state ( ∣G〉 ), the two bright exciton states ( $|\;X_{H/V}\;\rangle$ ), the dark exciton states ( $|\;D_{H/V}\;\rangle$ ), and the biexciton state ( ∣XX〉 ). Coupling induced by the magnetic field is abbreviated by $j_{\pm }$ . (**C**) Level scheme in the new eigenbasis including the magnetic field, displaying the mixed eigenstates $|\;X_{H/V}\;\rangle$ and $|\;D_{H/V}\;\rangle$ . Dashed downward arrows in (B) and (C) indicate optically active transitions and their coupling strength. (**D**) Results of the spectrally-resolved polarization map from a magneto-photoluminescence (PL) measurement at 3.4 T. The collection angle $\zeta^{C}$ specifies the projection onto a linear polarization state that is continuously varied in the range [−π,π] . Top: Linecuts at $\zeta^{C}=0$ (green, $X_{H}$ and $D_{H}$ ) and $\zeta^{C}=\frac{\pi }{2}$ (orange, $X_{V}$ ) and corresponding Gaussian fits (solid lines) showing three distinct emission lines. Inset: Polarization diagram for the three emission lines, extracted from the Gaussian fits. Black data points correspond to the total intensity of the bright states $|\;X_{H}\;\rangle$ and $|\;X_{V}\;\rangle$ . Solid lines are fits to a sinusoidal oscillation in intensity. For clarity not all data points are included in the figure. (**E**) Decay rate measured from $|\;D_{H}\;\rangle$ ( $\Gamma_{D}$ ) for increasing magnetic field strength $B_{x}$ . The inset shows the degree of mixing defined by the ratio of decay rates from $|\;D_{H}\;\rangle$ ( $\Gamma_{D}$ ) and $|\;X_{H}\;\rangle$ ( $\Gamma_{X}$ ). Solid lines are best fits to the theoretical model.

The direct optical manipulation of the dark state is enabled by a laser pulse sequence consisting of a transform-limited pulse and a pair of chirped pulses. All pulses are energetically tuned using 4f pulse shapers and chirps of ∓45 ps^2^ are introduced via reflection off a chirped volume Bragg grating (CVBG) (*29*, *52*). The quantum dot is hosted in a closed-cycle cryostat with a base temperature of 1.5 K, and optical activity of the dark state is controlled by a magnetic field of up to 4 T as indicated in Fig. 1A. The sample studied in this work contains GaAs/AlGaAs quantum dots and, together with the optical setup, is suitable for single–quantum dot studies. More details on the sample and the experimental methods are found in Materials and Methods and in (*35*, *53*).

The electronic system of the quantum dot consists of the ground state ∣G〉 , the single-exciton states and the biexciton state ∣XX〉 as indicated in Fig. 1B. Without the magnetic field, the energy eigenstates of the quantum dot can be divided into the bright states $|\;X_{H/V}\;\rangle$ , where the spins of electron and hole are opposite and dark states $|\;D_{H/V}\;\rangle$ with parallelly oriented spins. Bright states can be excited by linearly polarized light in horizontal (H) or vertical (V) polarization. With the same polarization, these exciton states couple to the biexciton ∣XX〉 , i.e., the two-photon emitting state (*4*, *5*, *54*). The dark states in the simplest picture are optically inaccessible. That means, once prepared, they would not decay optically. In reality, valence band mixing and Coulomb mixing to higher excited exciton states could lead to brightening of these dark states (*46*, *55*, *56*).

In our experiment, we induce a weak coupling between the bright and dark exciton states by a magnetic field $B_{x}$ in Voigt configuration ( $j_{\pm }\propto B_{x}$ in Fig. 1B). This leads to new eigenstates of the system, which we denote as $|\;X_{H/V}\;\rangle$ and $|\;D_{H/V}\;\rangle$ (see the Supplementary Text for a detailed description). In this basis, all states are optically coupled as indicated in Fig. 1C. Still, the coupling between the exciton states is quite different. For $|\;X_{H/V}\;\rangle$ the optical coupling matrix element is strong, resulting in a fast decay, while for $|\;D_{H/V}\;\rangle$ the optical coupling matrix element is rather weak with a slow decay rate. Hence, we keep the language from before and discriminate between bright $|\;X_{H/V}\;\rangle$ and dark exciton $|\;D_{H/V}\;\rangle$ states, although the new eigenstates $|\;D_{H/V}\;\rangle$ are not completely dark any more.

Initially, to identify the bright and dark states, a polarization-resolved magneto-photoluminescence (PL) measurement is performed as shown in Fig. 1D. For optical excitation, we use a 635-nm continuous wave laser source, and set the magnetic field strength to 3.4 T. Two bright emission lines around 1.56280 eV are identified, which vary out of phase as a function of collection polarization angle $\zeta^{C}$ . These lines correspond to emission from the transitions $|\;X_{H}\;\rangle \to |\;G\;\rangle$ and $|\;X_{V}\;\rangle \to |\;G\;\rangle$.

Besides the two bright exciton transitions, we observe an additional single dim emission line at 1.56265 eV. We attribute this emission to stem from the $|\;D_{H}\;\rangle \to |\;G\;\rangle$ transition based on its energetic position and its oscillatory behavior as function of the collection angle.

To further confirm the assignment of the states, we compute the degree of linear polarization $\text{DOLP}=\left(I_{x^{\prime }}-I_{y^{\prime }}\right)/\left(I_{x^{\prime }}+I_{y^{\prime }}\right)$ . Here $I_{x^{\prime }}$ is the maximum intensity and $I_{y^{\prime }}$ is intensity along an orthogonal axis. We obtain for the three transitions the values ${\text{DOLP}}_{X_{H}}=0.92\left(1\right)$ , ${\text{DOLP}}_{X_{V}}=0.94\left(1\right)$ , and ${\text{DOLP}}_{D_{H}}=0.73\left(1\right)$ , which is in agreement with similar work (*57*) and further corroborates our assumption of the identification of the dim line as a dark exciton. We note that the angle of maximum emission from $|\;D_{H}\;\rangle$ deviates from that of $|X_{H}\rangle$ by ≈−0.1 rad; see Fig. 1D.

The absence of emission from $|\;D_{V}\;\rangle$ suggests a highly anisotropic mixing behavior between the two polarization orientations which makes $|\;D_{V}\;\rangle$ non-addressable for this specific quantum dot in its orientation in the vector magnet. A strong preference for mixing along one polarization can occur for certain combinations of parameters, i.e., Landé *g*-factors ( $g_{\mathrm{ex}}$ and $g_{\mathrm{hx}}$ ), due to cancellations of the different parts (for an extended discussion see the “Theoretical model” section).

Using the information gained, the collection polarization is aligned with the quantum dot horizontal (vertical) polarization bases [light green (orange) line in Fig. 1D] by adjusting the collection angle $\zeta^{C}=0\left(\frac{\pi }{2}\right)$ . Note that the terms horizontal and vertical just refer to the linear orthogonal polarization states of the quantum dot.

To underline the discrimination of bright and dark states, we measure the decay rates of both $|\;X_{H}\;\rangle$ and $|\;D_{H}\;\rangle$ , denoted as $\Gamma_{X}$ and $\Gamma_{D}$ , respectively, in Fig. 1E. These rates were extracted from time-resolved photon-emission measurements and a double exponential fit to the data. As expected, the loss rate $\Gamma_{D}$ increases with increasing magnetic field strength. The inset shows the ratio of $\Gamma_{D}\;/\;\Gamma_{X}$ , reaching a maximum of ≈0.1 for the fields achievable in our experimental setup. Thus, a strong difference in lifetimes remains between bright and dark states, justifying that we can still distinguish between two types of excitons and call them bright $|\;X_{H/V}\;\rangle$ and dark $|\;D_{H/V}\;\rangle$ states. Magnetic field dependent measurements of $\Gamma_{X}$ are presented in the Supplementary Text (fig. S2) and are on the order of 5.5 (4.9) × 10^−3^ ps^−1^ for zero (3.5 T) magnetic field.

### Storage and retrieval using the dark exciton

#### Storage

**Fig. 2.**
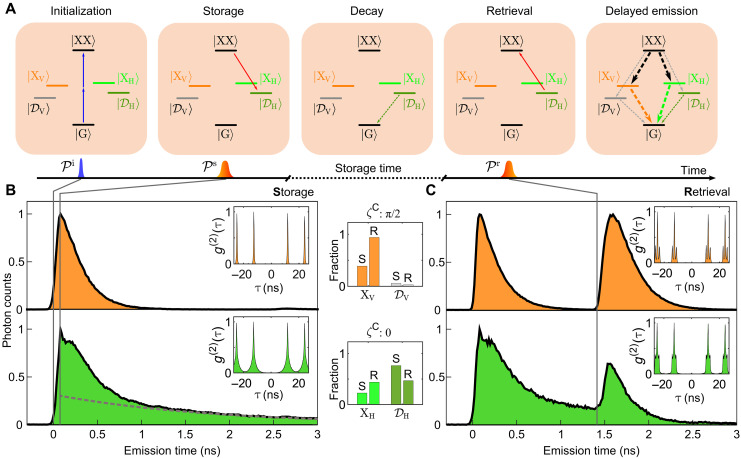
Time-resolved measurements and photon characteristics. (**A**) Sketches of the steps during the store-and-retrieve protocol. (**B**) Time-resolved photon emission during the storage sequence for vertical ( $\zeta^{C}=\frac{\pi }{2}$ , orange) and horizontal polarization ( $\zeta^{C}=0$ , green). A dashed gray line indicates the long-timescale decay from $|\;D_{H}\;\rangle$ . The insets show the second-order autocorrelation traces [ $g^{\left(2\right)}\left(\tau \right)$ ]. (**C**) The same as for (B) but including the retrieval pulse $P^{r}$ . In the center panels, we compare the integrated photon counts assigned to emission energies from $|\;X_{H/V}\;\rangle$ and $|\;D_{H/V}\;\rangle$ when switching from storage (S) to retrieval (R).

Having identified the dark exciton, the aim is to optically control its state in a storage and retrieval sequence of optical pulses as indicated in Fig. 2A. The full sequence comprises of a series of laser pulses with variable time delay between them. In this sequence, the storage process consists of the initialization ( $P^{i}$ ) and storage pulse ( $P^{s}$ ). The first pulse $P^{i}$ brings the system from its ground to the biexciton state via two-photon excitation (*4*, *5*). The following storage pulse $P^{s}$ prepares the system in the dark exciton state $|\;D_{H}\;\rangle$ starting from the biexciton. The preparation of the dark exciton is achieved by using a horizontally polarized, negatively chirped [group delay dispersion (GDD) = −45 ps^2^] laser pulse, inducing an adiabatic evolution of states, similar to the suggestion in (*48*).

The storage sequence is monitored via the time-resolved photon emission under the application of the two pulses as shown in Fig. 2B. We note that all states are now optically active with differently strong dipole moments, such that radiative emission takes places at all times. We take two sets of data: (orange) emission of $|\;X_{V}\;\rangle$ at $\zeta^{C}=\frac{\pi }{2}$ and $B_{x}=2$ T and (green) emission from $|\;X_{H}\;\rangle$ and $|\;D_{H}\;\rangle$ at $\zeta^{C}=0$ and $B_{x}=3.4$ T. The action of the two pulses is marked by vertical lines in the figure.

Induced by the initialization pulse, the biexciton becomes occupied, resulting in a cascaded decay behavior following $|\;\text{XX}\rangle \to |\;X_{H/V}\rangle \;/\;|\;D_{H/V}\;\rangle \to |\;G\;\rangle$ accompanied by an immediate rise in photon counts.

At ≈0.07 ns, the storage pulse $P^{s}$ is applied. This interrupts the decay of the biexciton into the exciton states and, therefore, the photon emission from these transitions. Detecting the vertical polarization (orange), after the storage pulse, we observe only the emission $|\;X_{V}\;\rangle \to |\;G\;\rangle$ indicated by an abrupt transition to single exponential decay, signaling the depopulation of ∣XX〉 induced by $P^{s}$ . While in an ideal storage process this emission would vanish, the decay during the initialization pulse up to the storage pulse already leads to an occupation of $|\;X_{V}\;\rangle$.

In the case of horizontal detection (green) the behavior is different and with the storage pulse, a double exponential decay from $|\;X_{H}\;\rangle$ and $|\;D_{H}\;\rangle$ sets in. In addition, we find a sharp feature during the storage pulse $P^{s}$ . Here, a transient occupation during the pulse of the bright exciton $|\;X_{H}\;\rangle$ leads to a strong increase of photon counts during $P^{s}$ . Imperfections in the preparation protocol lead to population remaining in both the bright exciton and the biexciton, yielding the shoulder following the sharp feature at ≈0.25 ns.

Remembering that the dark exciton eigenstate $|\;D_{H}\;\rangle$ has a finite lifetime (cf. Fig. 1E) and eventually decays, the slow exponential decay then stems from the dark exciton with a rate of $\Gamma_{D}$ ≈ 0.56 ns^−1^ indicated as a dashed gray line. Comparing the two cases (orange and green) already signals that we have prepared the dark exciton $|\;D_{H}\;\rangle$.

#### Retrieval

After a storage time long enough for the states ∣XX〉 and $|\;X_{H/V}\;\rangle$ to fully decay [ $\approx 1.3\;\text{ns}>3*{\left(\Gamma_{X}\right)}^{-1}$ ], we apply a positively (GDD = 45 ps^2^) chirped laser pulse to retrieve the dark exciton population and bring the system back into the biexciton state. From the biexciton state, a cascaded emission into the ground state takes place and is recorded in the photon emission.

In Fig. 2C, we show the data recorded for the whole protocol. For both collection angles, we see that, by the retrieval pulse, again, photon emission is triggered.

It is important to compare the two shapes of emission peaks at the storage and retrieval steps for vertical collection polarization (orange). In the storage step, we find mostly a single exponential decay starting abruptly after $P^{s}$ arrives (see Fig. 2B). In contrast, in the retrieval step, there is a rise followed by a smooth transition to an exponential decay. This is also obvious in the widths of the two patterns. This behavior is typical for a cascaded decay, where the exciton is fed by the biexciton while simultaneously decaying into the ground state.

The area under the second peak is set by the storage sequence, which determines the amount of population to be stored. As such, the second peak can be adjusted by the time difference between $P^{i}$ and $P^{s}$ because the decay between the pulses is related to the storable population. We observe that, in the vertical case, the second emission peak is about the same height as the first emission peak, while, in the horizontal case, the second emission peak is less pronounced.

After the arrival of $P^{r}$ , i.e., at timescales longer than 1.5 ns, the dynamics is mostly governed by the decay via the bright exciton states. A negligible response at long timescales, i.e., after 2.5 ns, hints toward the small but finite optical activity of the dark exciton decay channel for horizontal (green) polarization.

### Photon counts and single-photon character

In the center of Fig. 2, we quantify the amount of photons emitted during the storage (S) and retrieval (R) parts by integrating the photon counts and discriminating them by their emission energy, indicated by $X_{H/V}$ and $D_{H/V}$ . We note that only a negligible contribution from $|\;D_{V}\;\rangle$ is observed (a detailed explanation is shown in the “Theoretical model” section).

In the vertical (orange) case, the photon counts are normalized to the total photon emission during the full sequence, where we find that almost all photons are detected on the bright exciton line $X_{V}$ . In the green case, i.e., for horizontal collection angle, the photon counts are normalized to the total emission during the storage sequence. Here, the emission originates mostly from the dark exciton $|\;D_{H}\;\rangle$.

Note that the initial decay after $P^{i}$ but before $P^{s}$ arrives leads to still a finite emission from the bright exciton $|\;X_{H}\;\rangle$ . After the full sequence, the photon counts are almost equally distributed between the dark and bright exciton states. This is indicated by the green bar plot in the center of Fig. 2. By adjusting the parameters, this ratio could also be adjusted for a higher-efficiency preparation of $|\;D_{H}\;\rangle$.

To characterize the nature of emission, we also recorded the second-order autocorrelation traces [ $g^{\left(2\right)}\left(\tau \right)$ ] for all four cases. We find that, in all cases, $g^{\left(2\right)}\left(0\right)$ is vanishing, proving the single-photon nature for both the recorded polarizations. In particular, this certifies that, for the full sequence, the photon storage and retrieval have been successful.

### Theoretical analysis

To understand the sequence to store and retrieve in and out of the dark state as well as the action of the chirped laser pulses, we performed numerical simulations of dynamics of the quantum dot states. For this, we set up a quantum dot model of the six electronic states and include the coupling to the external magnetic field as well as the pulsed optical driving. For the corresponding Hamiltonians, we refer the reader to the “Theoretical model” section.

The full sequence comprises a series of laser pulses with the arrival times $t_{0}$ and the width τ . The pulses have different frequencies $\omega_{P}$ and can be of different polarizations $e_{P}$ . Key to the protocol is allowing the storage and retrieval pulses to be chirped with the chirp coefficient a , such that the pulses read

$$\begin{array}{c} P∼e_{P}\text{exp}\left[-\frac{{\left(t-t_{0}\right)}^{2}}{2\tau^{2}}\right]\times \\ \text{exp}\left\{-i\left[\omega_{P}+\frac{a\left(t-t_{0}\right)}{2}\right]\left(t-t_{0}\right)\right\} \end{array}$$ (1)

More details on the driving fields are found in the Supplementary Text.

In addition, we account for radiative decay by a Lindblad operator ℒ via the rates $\Gamma_{i}$ . We introduce the rates $\Gamma_{X}$ and $\Gamma_{\text{XX}}$ in the bare state system (see Fig. 1B) that describe the decays of the bright states $|\;X_{H/V}\;\rangle \to |\;G\;\rangle$ and $|\;\text{XX}\;\rangle \to |\;X_{H/V}\;\rangle$ , respectively, where the dark states do not couple to the light field and, therefore, do not decay. In the eigenstate picture (see Fig. 1C), all transitions have a finite dipole moment, and, accordingly, now all transitions are accompanied by a decay. Note that we perform all the numerical simulations in the bare state basis.

We additionally account for the coupling to the phononic environment of the quantum dot. Phonons are known to be the major source of decoherence in quantum dot dynamics (*22*, *23*). We include the phonons on a microscopic level and solve the occurring many-body problem with a process tensor matrix product operator method for a numerically exact description (*50*, *51*). The simulation parameters are found in the Supplementary Text (table S1).

**Fig. 3.**
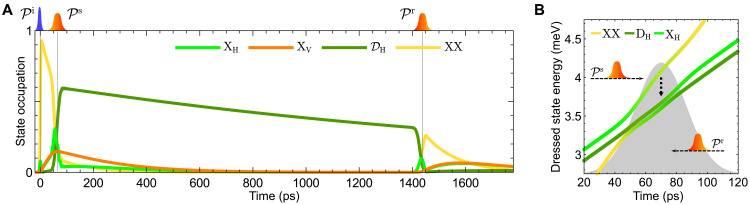
Simulation of the quantum dot dynamics. (**A**) Dynamics of the state occupations of the bright states $|\;X_{H/V}\;\rangle$ , the dark exciton state $|\;D_{H}\;\rangle$ , and the biexciton state ∣XX〉 under the pulse sequence displayed on top. (**B**) Dressed state analysis of the system under the influence of $P^{s}$ ( $P^{r}$ ) if read from right to left. The states $|\;X_{V}\;\rangle$ , $|\;D_{V}\;\rangle$ , and ∣G〉 are effectively not influenced by $P^{s}$ ( $P^{r}$ ) and are, therefore, excluded. Colors indicate the instantaneous eigenstate’s overlap to the undisturbed bare states shown in Fig. 1B. The dotted downward arrow illustrates phonon relaxation processes.

The results of the numerical calculations are shown in Fig. 3A. The occupations of the quantum dot states $|\;X_{H/V}\;\rangle$ , $|\;D_{H}\;\rangle$ , and ∣XX〉 are displayed under the action of the laser pulse sequence composed of $P^{i}$ , $P^{s}$ , and $P^{r}$ shown on top. Initially, only the ground state is occupied, such that all displayed occupations are zero. The first pulse $P^{i}$ leads to an occupation of the biexciton visible as strong rise of the biexciton state occupation. It is followed by the decay into the two bright excitons $|\;X_{H/V}\;\rangle$.

The storage pulse $P^{s}$ brings most of the biexciton occupation into the dark exciton state $|\;D_{H}\;\rangle$ . During the pulse, we see a transient occupation of the horizontal bright exciton $|\;X_{H}\;\rangle$ . This transient occupation results in the sharp feature observed in Fig. 2B. The other bright exciton $|\;X_{V}\;\rangle$ is unaffected by the pulse.

After the storage pulse, a decay behavior follows: On the one hand, there is still some biexciton occupation left, which decays mostly via the bright exciton states, which, in turn, decay into the ground state. The decay of the dark exciton state $|\;D_{H}\;\rangle$ occurs much slower. After half the storage time, i.e., around 700 ps, there is no occupation left in the bright states, while about 75% of the dark exciton occupation after the pulse remains. After the full storage time, we apply the retrieval pulse $P^{r}$ , which switches the dark exciton occupation back to the biexciton state. From there, the cascaded decay ( $|\;\text{XX}\;\rangle \to |\;X_{H/V}\;\rangle \to |\;G\;\rangle$ ) takes place.

To understand the action of the chirped pulses $P^{s}$ and $P^{r}$ , which are key to our storage and retrieval protocol, we consider their action in the dressed state picture (*58*). The dressed state energies (i.e., the instantaneous eigenenergies of the coupled system) of the participating states in the rotating frame of $P^{s}$ are plotted in Fig. 3B. Due to the rotating frame definition of the linearly chirped laser pulse, the dressed-state energies are linearly rising with time and falling if read from right-to-left for positive chirp. The colors represent the overlap of the instantaneous eigenstates to the undisturbed bare states ∣XX〉 (yellow), $|\;X_{H}\;\rangle$ (light green), and $|\;D_{H}\;\rangle$ (dark green). The dressed state corresponding to the biexciton state before and after the pulse has a steeper slope by a factor of 2, due to its energy being about twice that of the single-exciton state. For the dynamics induced by storage and retrieval pulses, the remaining states do not contribute and are, therefore, not shown.

Because $P^{r}$ differs only in the sign of chirp from $P^{s}$ , reading Fig. 3B from right to left reveals the action of $P^{r}$ : Reversing the chirp effectively corresponds to inverting the pulse in time. During the action of the pulse, the energies shift apart and multiple anti-crossings occur, connected to an adiabatic evolution of the states. This means that, during the dynamics, only little population is swapped between the dressed states. The population transfer in the bare state picture is mostly governed by the change in mixing of the dressed states, here visualized as the change of line color. Via the adiabatic evolution during the pulses $P^{s}$ (top arrow) and $P^{r}$ (bottom arrow), the following states are connected:

1. $|\;X_{H}\;\rangle ⇌|\;\text{XX}\;\rangle$

2. $|\;D_{H}\;\rangle ⇌|\;X_{H}\;\rangle$

3. $|\;\text{XX}\;\rangle ⇌|\;D_{H}\;\rangle$

For our protocol, that means that we use the lowest dressed state going from $|\;\text{XX}\;\rangle \to |\;D_{H}\;\rangle$ (yellow to dark green) with $P^{s}$ and reverse with $P^{r}$ to transfer the population between the biexciton and the dark state. Note that, during the evolution, this lowest dressed state is mainly characterized by $|\;X_{H}\;\rangle$ (light green segment in the middle), which in the temporal dynamics shows as a transient population of this state.

Phonons have little effect on the overall population dynamics, which is confirmed by simulations with and without phonons (see Supplementary Text, fig. S1). This can mainly be deduced from the fact that the evolution along the lowest dressed state at low temperatures does not induce phonon transitions (*59*). Possible phonon transitions would most efficiently couple the upper dressed state to the lower dressed states, as indicated by the vertical dashed arrow. This effectively benefits the protocol, as a finite population present in the corresponding upper dressed state can be transferred to the lower dressed states by phonon-mediated processes.

### Performance analysis

In reality, the successful preparation of $|\;D_{H}\;\rangle$ is dictated by the parameters of $P^{s}$ and limited by finite pulse durations, temporal separation of the pulses, and the decay from ∣XX〉.

Therefore, we investigate the preparation efficiency of $|\;D_{H}\;\rangle$ by studying three parameters of the storage pulse $P^{s}$:

1. Time delay between $P^{i}$ and $P^{s}$ : Δt

2. Polarization of $P^{s}$ : $\zeta^{P}$

3. Energetic detuning of $P^{s}$ with respect to the transition $|\;\text{XX}\;\rangle \to |\;X_{H}\;\rangle$ : ΔE

**Fig. 4.**
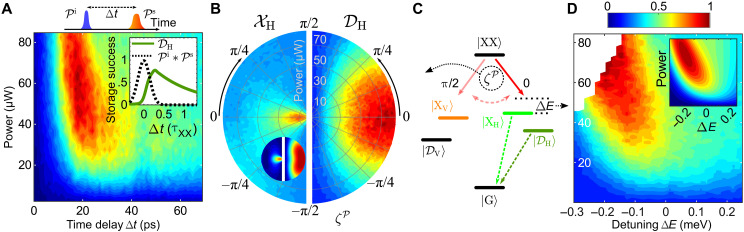
Dark state preparation characterization. (**A**) Photon counts recorded from $|D_{H}\rangle$ for a variation of the temporal separation of $P^{i}$ and $P^{s}$ ( Δt ) and the pulse power of $P^{s}$ . The inset shows a numerical calculation of the storage fidelity at optimum power in units of the lifetime of ∣XX〉 ( $\tau_{\text{XX}}$ ) alongside the normalized convolution of $P^{i}$ and $P^{s}$ ( $P^{i}*P^{s}$ ). (**B**) Photon emission from $|X_{H}\rangle$ (left) and $|D_{H}\rangle$ (right) for varying angle of linear polarization ( $\zeta^{P}$ ) of $P^{s}$ . Pulse power is varied along the radial axis and for symmetry reasons only data in the range $\zeta^{P}=\left[-\frac{\pi }{2},\frac{\pi }{2}\right]$ is shown. The inset shows a numerical calculation of the idealized process excluding decays. (**C**) Illustration of the parameters investigated in (B) and (D). (**D**) The same as for (A) but for varying detuning of $P^{s}$ ( ΔE ) relative to the emission energy $|\;\text{XX}\;\rangle \to |\;X_{H}\;\rangle$ . The inset shows the numerical calculation of the idealized process, excluding decays. Photon counts shown in (A), (B), and (D) are normalized to their respective maximum, while data extracted from the simulations, presented in the insets, show the preparation probability. All measurements were performed at a magnetic field strength of $B_{x}=3.4$ T.

In Fig. 4, we summarize the results. The efficiency of the dark state preparation is monitored by the integrated photon counts at the corresponding emission energy (see Fig. 1D). All measurements are performed at a magnetic field strength of $B_{x}=3.4$ T.

Time delay: The storage step relies on the application of a storage pulse $P^{s}$ after initial preparation of ∣XX〉 via a two photon resonant initialization pulse $P^{i}$ . The storage pulse $P^{s}$ needs to be negatively chirped to induce the adiabatic passage from ∣XX〉 to $|\;D_{H}\;\rangle$ . As a consequence, the transition into $|\;D_{H}\;\rangle$ cannot happen instantaneously but requires a finite pulse length (cf. eq. S1 in the Supplementary Text). Consequently, this imposes a finite time delay Δt between $P^{i}$ and $P^{s}$ , during which the decay from ∣XX〉 after $P^{i}$ lowers the preparation fidelity of $|\;D_{H}\;\rangle$.

In Fig. 4A, we present a two-dimensional map of the measured $|\;D_{H}\;\rangle$ population as a function of the time delay Δt between $P^{i}$ and $P^{s}$ and power of $P^{s}$ . For all powers of $P^{s}$ , a maximum population of $|\;D_{H}\;\rangle$ is found at Δt ≈ 20 ps.

To understand the limits imposed by a finite ∣XX〉 lifetime ( $\tau_{\text{XX}}$ ) and pulse durations, we simulate the storage success given by the occupation of $|\;D_{H}\;\rangle$ . For this, we set the $P^{s}$ power to its optimal value and study the dependence on Δt . We calculate the temporal overlap of $P^{i}$ and $P^{s}$ as the normalized convolution of both field amplitudes ( $P^{i}*P^{s}$ ) and present the results in units of $\tau_{\text{XX}}$ . The inset in Fig. 4A shows that efficient population of $|\;D_{H}\;\rangle$ can only be achieved when Δt is sufficiently large such that $P^{i}*P^{s}\ll 1$ , meaning the pulses should not overlap substantially. At larger Δt , the fidelity is then further reduced by a finite $\tau_{\text{XX}}$.

Polarization: In Fig. 4B, we study the preparation efficiency of $|\;D_{H}\;\rangle$ depending on the angle of linear polarization $\zeta^{P}$ and optical power of $P^{s}$ (as sketched in Fig. 4C). For low powers, the pulse sequence $P^{i}+P^{s}$ leads to an increase of emission from $|\;X_{H}\;\rangle$ compared to pure two-photon resonant excitation (power of $P^{s}=0$ , innermost data points), if the polarization is aligned parallel to the transition $|\;\text{XX}\;\rangle \to |\;X_{H}\;\rangle$ ( $\zeta^{P}=0$ ). In the case of orthogonal polarization ( $\zeta^{P}=\pm \frac{\pi }{2}$ ), a suppression of emission is observed; see left side of Fig. 4B. This response has been observed before, e.g., in (*35*). While this process shows a strong dependence on $\zeta^{P}$ , we find that the preparation of $|\;D_{H}\;\rangle$ is less sensitive to $\zeta^{P}$ and happens at higher powers (right side of Fig. 4B). We attribute this lowered sensitivity to the anisotropic mixing of $|\;X_{H}\;\rangle ↔|\;D_{H}\;\rangle$ and $|\;X_{V}\;\rangle ↔|\;D_{V}\;\rangle$ , effectively favoring a transition into $|\;D_{H}\;\rangle$ at higher pulse powers.

Detuning: In Fig. 4D, we present the recorded photon emission from $|\;D_{H}\;\rangle$ when the central energy of $P^{s}$ is tuned with respect to the transition energy $|\;\text{XX}\;\rangle \to |\;X_{H}\;\rangle$ ( ΔE , see Fig. 4C) and its power is varied. We compare the recorded emission to numerical simulations of $|\;D_{H}\;\rangle$ population presented in the inset and find an optimum value for ΔE of about −0.2 meV or ≈1.5590 eV absolute energy in both cases. Because of the robustness of the adiabatic passage, a small detuning of the central frequency maintains a high final dark state population (*53*, *60*). This feature can also be beneficial in a multilevel system (*61*).

## DISCUSSION

In summary, we have designed and demonstrated a method of storing and retrieving population using the dark state in a semiconductor quantum dot. We have also provided an in-depth understanding of our quantum emitter system with theoretical simulations. With the usage of the external magnetic field and the chirped laser pulses, the system stays within the ground state manifold of the quantum dot, and the optical control processes are coherent. As our protocol relies on pulses detuned from the exciton energy, it offers the advantage of simple spectral filtering being efficient for laser light suppression. Using the dark exciton could improve several protocols to generate time-bin entanglement states (*6*, *44*, *45*, *62*) or photonic cluster states (*63*, *64*). Our results help leverage the versatile quantum dot state manifold including the optically dark exciton states and further expand the possibility of using quantum dots for quantum communication.

## MATERIALS AND METHODS

### Experimental methods

The quantum dot is hosted in a closed-cycle helium cryostat (ICEOxford) with a base temperature of about 1.5 K and a superconducting vector magnet system (up to 4-T absolute value). We excite the quantum dot and collect photons from the top through a cold objective (numerical aperture of 0.81, attocube systems AG), using a cross-polarization measurement setup for scattered laser light suppression. The collection polarization basis is chosen linear and can be rotated by means of a half-wave plate (HWP). Collected photons are sent through a homebuilt monochromator based on narrowband notch filters [BNF-805-OD3, full width at half maximum (FWHM) of 0.3 nm, Optigrate], which is set up to collect single-photon emission and spectrally block scattered laser reflection. Measurements on spectral composition were performed using a single-photon sensitive spectrometer (Acton SP-2750, Roper Scientific) equipped with a liquid nitrogen cooled charge-coupled device camera (Spec10 CCD, Princeton Instruments). Time-sensitive measurements were carried out on superconducting nanowire single-photon detectors (Eos, Single Quantum) connected to a time-tagging module (Time Tagger Ultra, Swabian Instruments). The overall time jitter of the single-photon measurement apparatus was 20 ps.

The sequence of excitation pulses is created by sending a single ≈2-ps FWHM long pulse (Tsunami 3950, Spectra-Physics) through two individual 4f pulse shapers with variable slit positions. We prepare one pulse to be two-photon excitation (TPE) resonant with a spectral bandwidth of ≈0.2-nm FWHM. The other pulse is spectrally centered around the biexciton emission wavelength and also of ≈0.2-nm FWHM. We split this pulse at a 50:50 beam splitter (BS) and send both onto a CVBG (Optigrate). This prepares both pulses to carry ∓45 ps^2^ of spectral chirp, depending of the direction of reflection. We delay the positively chirped pulse by ≈1.3 ns before recombining both on a second BS. The set of chirped pulses are delayed with respect to the TPE resonant pulse by a fiber-optic delay line (ODL-300, OZ Optics). Recombination of both excitation paths happens at a 90:10 BS close to the cryostat entrance window. The polarization of the chirped pulses can be arbitrarily chosen via the means of a HWP/quarter wave plate combination while the polarization of the TPE pulse is fixed to be orthogonal to the collection polarization via a polarizing BS. A detailed drawing of the experimental setup can be found in the Supplementary Text (fig. S3).

### Theoretical model

The Hamiltonian used to model the system dynamics can be written in the dipole and rotating wave approximation, similar to our previous work (*35*, *53*) as

$$\hat{H}={\hat{H}}^{\text{system}-\text{fields}}+{\hat{H}}^{\text{phonon}}$$ (2)

where ${\hat{H}}^{\text{system}-\text{fields}}$ treats the quantum dot system and its interaction with electromagnetic fields and reads as

$${\hat{H}}^{\text{system}-\text{fields}}={\hat{H}}^{\text{QD}}+{\hat{H}}^{\mathrm{Bx}}+{\hat{H}}^{L}$$ (3)

The individual parts are described in the following. We model the quantum dot as a six-level system in the linear polarization basis consisting of the ground state ∣G〉 , two bright excitons of horizontal ( $|\;X_{H}\;\rangle$ ) and vertical ( $|\;X_{V}\;\rangle$ ) polarization, neighboring dark excitons ( $|\;D_{H}\;\rangle$ and $|\;D_{V}\;\rangle$ ), and the biexciton state ∣XX〉 (see Fig. 1B).

The ground-state energy is set to zero, while the two bright excitons are separated by a fine-structure splitting $\delta_{X}$ , such that $E_{X}^{H/V}=\hbar \omega_{X}\pm \frac{\delta_{X}}{2}$ . The biexciton has a binding energy $E_{B}$ such that $E_{\text{XX}}=2\hbar \omega_{X}-E_{B}$ . The dark states are treated similar to the bright states and have a fine structure splitting $\delta_{D}$ , such that $E_{D}^{H/V}=\hbar \omega_{D}\pm \frac{\delta_{D}}{2}$ . The quantum dot Hamiltonian then reads

$$\begin{array}{cc} {\hat{H}}^{\text{QD}} & =E_{X}^{H}|\;X_{H}\rangle \langle X_{H}\;|+E_{X}^{V}|\;X_{V}\rangle \langle X_{V}\;|\\ & +E_{D}^{H}|\;D_{H}\rangle \langle D_{H}\;|+E_{D}^{V}|\;D_{V}\rangle \langle D_{V}\;|\\ & +E_{\text{XX}}|\;\text{XX}\rangle \langle \text{XX}\;| \end{array}$$ (4)

Coupling to an in-plane magnetic field in Voigt configuration is included in ${\hat{H}}^{\text{B}\text{x}}$ and introduces a mixing between bright states and their dark counterparts via

$$\begin{array}{ll} {\hat{H}}^{\mathrm{Bx}} & =-\mu_{B}\frac{B_{x}}{2}\left(g_{hx}+g_{ex}\right)|\;X_{H}\rangle \langle D_{H}\;|\\ & -\mu_{B}\frac{B_{x}}{2}\left(g_{hx}-g_{ex}\right)|\;X_{V}\rangle \langle D_{V}\;|+h.c. \end{array}$$ (5)

Here, $g_{ex}\left(g_{hx}\right)$ denote the Landé *g*-factors and the coupling of the electron (hole) to the magnetic field, while $\mu_{B}$ is the Bohr magneton. For better readability the coupling terms $-\mu_{B}\frac{B_{x}}{2}\left(g_{hx}\pm g_{ex}\right)$ are abbreviated by $j_{\pm }$ in Fig. 1C. The asymmetry of $j_{+}$ and $j_{-}$ allows for strong anisotropic mixing if $|\;g_{ex}\;|\approx |\;g_{hx}\;|$ as is the case for the quantum dot used in this work, i.e., $j_{+}\gg j_{-}$.

The laser driving fields are coupled to the quantum dot via

$$\begin{array}{cc} {\hat{H}}^{L} & =\Omega \left(\Delta t,\omega_{P},\Theta ,\text{GDD},e_{L}\right)\cdot \\ & [e_{H}\left(\;|\;X_{H}\rangle \langle G|+|\;\text{XX}\rangle \langle X_{H}\;|\;\right)+\\ & e_{V}\left(\;|\;X_{V}\rangle \langle G\;|+|\;\text{XX}\rangle \langle X_{V}\;|\;\right)+h.c.] \end{array}$$ (6)

Here, $e_{H}$ and $e_{V}$ are vectors of unit length and represent the vectorial overlap of the horizontal and vertical dipole moments to the laser polarization ( $e_{L}$ ) via the dot product (see also Fig. 1D).

The coupling term $\Omega \left(\Delta t,\omega_{P},\Theta ,\text{GDD},e_{L}\right)$ is treated as classical chirped laser fields of Gaussian shape, see eq. S1 and the “Quantum dot sample” section.

We consider radiative decay and losses by a Lindblad operator L. For a density matrix $\hat{\rho }$ , a rate Γ and operators $\hat{O}$ , the Lindblad operator takes the form

$$L_{\Gamma ,\hat{O}}\left[\hat{\rho }\right]=\Gamma \left(\hat{O}\;\hat{\rho }\;{\hat{O}}^{†}-\frac{1}{2}\left\{\hat{\rho },\;{\hat{O}}^{†}\;\hat{O}\right\}\right)$$ (7)

with {.,.} indicating the anticommutator. We incorporate radiative decays such that the Lindblad superoperators read

$$\begin{array}{c} \begin{array}{ll} L\left[\hat{\rho }\right] & ≔L_{\Gamma_{X},|G\rangle \langle X_{H}|}\left[\hat{\rho }\right]+L_{\Gamma_{X},|G\rangle \langle X_{V}|}\left[\hat{\rho }\right]\\ & +L_{\Gamma_{\text{XX}},|X_{H}\rangle \langle \text{XX}|}\left[\hat{\rho }\right]+L_{\Gamma_{\text{XX}},|X_{V}\rangle \langle \text{XX}|}\left[\hat{\rho }\right] \end{array} \end{array}$$ (8)

The rates $\Gamma_{X}$ and $\Gamma_{\text{XX}}$ describe the decays $|X_{H}\rangle \;/\;|X_{V}\rangle \to |G\rangle$ and $|\;\text{XX}\;\rangle \;\to |\;X_{H}\;\rangle \;/\;|\;X_{V}\;\rangle$ , respectively, and are indicated by downward dashed arrows in Fig. 1B. Note that, for the bare states, no decay from the dark states is contained in the model ( $\Gamma_{D}=0$).

Only the inclusion of ${\hat{H}}^{\mathrm{Bx}}$ leads to a mixing of bright and dark states and the formation of new eigenstates in the time-independent Hamiltonian ${\hat{H}}^{\text{QD}}+{\hat{H}}^{\mathrm{Bx}}$ . Therefore, we introduce the new states $|\;X_{H}\;\rangle$ , $|\;D_{H}\;\rangle$ , $|\;X_{V}\;\rangle$ , and $|\;D_{V}\;\rangle$ as the eigenstates of ${\hat{H}}^{\text{QD}}+{\hat{H}}^{\mathrm{Bx}}$ . These states inherit properties of their respective parent states, including the coupling to the driving fields and radiative decay via photon emission, resulting in partially bright dark states as depicted in Fig. 1C. A decomposition of these states in terms of the original bare states and the new eigenenergies can be found in the “Quantum dot sample” section.

Additionally, we model dissipation via the coupling to longitudinal acoustic phonons by using the deformation potential coupling. With ${\hat{b}}_{k}$ ( ${\hat{b}}_{k}^{†}$ ) as annihilation (creation) operator of a phonon mode k with frequency $\omega_{k}$ , the phonon coupling Hamiltonian reads as

$${\hat{H}}^{\text{phonon}}=\hbar \sum_{k}\omega_{k}{\hat{b}}_{k}{\hat{b}}_{k}^{†}+\hbar \sum_{k,S}\left(\gamma_{k}^{S}{\hat{b}}_{k}^{†}+{\gamma_{k}^{S}}^{*}{\hat{b}}_{k}\right)|S\rangle \langle S|$$ (9)

Here, each phonon mode k couples with a coupling constant $\gamma_{k}^{S}$ to a quantum dot state ∣S〉 , where $S\in \left\{\;X_{H},X_{V},D_{H},D_{V},\text{XX}\;\right\}$ . The coupling constant $\gamma_{k}^{S}$ with its including material parameter are chosen to be the same as in (*65*) and our previous works (*35*, *53*).

### Quantum dot sample

The sample used contains GaAs/AlGaAs quantum dots obtained by the Al-droplet etching method (*14*) and was grown by molecular beam epitaxy. The quantum dots are embedded in the center of a λ-cavity placed between a bottom (top) distributed Bragg reflector consisting of nine (*2*) pairs of ^λ^/_4_-thick Al_0.95_Ga_0.05_As/Al_0.20_Ga_0.80_As layers with respective thicknesses of 69/60 nm. The quantum dots are placed between two ^λ^/_2_-thick Al_0.33_Ga_0.67_As layers. The quantum dot growth process starts by depositing 0.5 equivalent monolayers of Al in the absence of arsenic flux, which results in the self-assembled formation of droplets. During exposure to a reduced As flux, such droplets locally etch the underlying Al_0.33_Ga_0.67_As layer, resulting in ≈9-nm-deep and ≈60-nm-wide nanoholes on the surface. Then, the nanoholes are filled with GaAs by depositing ≈1.1 nm of GaAs on the surface, followed by an annealing step of 45 s. The temperature used for the etching of the nanoholes was 600°C. The droplet self-assembly process results in quantum dots with random position and a surface density of about 2 × 10^7^ cm^−2^, suitable for single–quantum dot spectroscopy. We note that the same sample was also used in our previous works (*35*, *53*).
