# Supplementary material for: Keeping the photon in the dark: Enabling quantum dot dark state control by chirped pulses and magnetic fields
Source: Sci Adv. 2025 Jul 9;11(28):eadu4261. doi: 10.1126/sciadv.adu4261 (PMC13155481; doi:10.1126/sciadv.adu4261)
Supplement: Supplementary file 1 — Supplementary Text Figs. S1 to S3 Table S1 [file sciadv.adu4261_sm.pdf]

Science Advances
AAAS

Supplementary Materials for

Keeping the photon in the dark: Enabling quantum dot dark state control by chirped pulses and magnetic fields

Florian Kappe *et al.*

Corresponding author: Florian Kappe, florian.kappe@uibk.ac.at

Sci. Adv. **11**, eadu4261 (2025)
DOI: 10.1126/sciadv.adu4261

This PDF file includes:

Supplementary Text
Figs. S1 to S3
Table S1

Supplementary Text

Quantum dot states in Voigt configuration

As described in the main text we utilize an in-plane magnetic field in Voigt configuration to introduce a mixing of the initial bare bright states $|X_H\rangle$ and $|X_V\rangle$ with the initial dark states $|D_H\rangle$ and $|X_V\rangle$. This leads to the formation of new eigenstates ($|\mathcal{X}_H\rangle$, $|\mathcal{X}_V\rangle$, $|\mathcal{D}_H\rangle$ and $|\mathcal{D}_V\rangle$) and new eigenergies via the diagonalisation of the time independent Hamiltonian $\hat{H}^{QD} + \hat{H}^{Bx}$. Since $\hat{H}^{Bx}$ does not introduce any mixing on them and for better readability the states $|G\rangle$ and $|XX\rangle$ are omitted and we restrict our self to the four dimensional subspace spanned by single excitonic states:

$$\begin{pmatrix} E_X^H & 0 & j_+ & 0 \\ 0 & E_X^V & 0 & j_- \\ j_+ & 0 & E_D^H & 0 \\ 0 & j_- & 0 & E_D^V \end{pmatrix} \to \begin{pmatrix} E_{\mathcal{X}}^H & 0 & 0 & 0 \\ 0 & E_{\mathcal{X}}^V & 0 & 0 \\ 0 & 0 & E_{\mathcal{D}}^H & 0 \\ 0 & 0 & 0 & E_{\mathcal{D}}^V \end{pmatrix}$$

Here $j_\pm = -\mu_B \frac{B_x}{2}(g_{hx} \pm g_{ex})$ introduces the magnetic field dependent mixing. The new eigenenergies read as:

$$\begin{aligned} E_{\mathcal{X}}^H &= \frac{1}{2}(+\sqrt{(E_X^H - E_D^H)^2 + 4j_+^2} + E_X^H + E_D^H) \\ E_{\mathcal{X}}^V &= \frac{1}{2}(+\sqrt{(E_X^V - E_D^V)^2 + 4j_-^2} + E_X^V + E_D^V) \\ E_{\mathcal{D}}^H &= \frac{1}{2}(-\sqrt{(E_X^H - E_D^H)^2 + 4j_+^2} + E_X^H + E_D^H) \\ E_{\mathcal{D}}^V &= \frac{1}{2}(-\sqrt{(E_X^V - E_D^V)^2 + 4j_-^2} + E_X^V + E_D^V). \end{aligned}$$

Decomposing the new mixed states in terms of the bare states reads:

$$\begin{aligned} |\mathcal{X}_H\rangle &= \frac{1}{N}(\frac{E_X^H - E_D^H + \sqrt{(E_X^H - E_D^H)^2 + 4j_+^2}}{2j_+}|X_H\rangle + |D_H\rangle) \\ |\mathcal{X}_V\rangle &= \frac{1}{N}(\frac{E_X^V - E_D^V + \sqrt{(E_X^V - E_D^V)^2 + 4j_-^2}}{2j_-}|X_V\rangle + |D_V\rangle) \\ |\mathcal{D}_H\rangle &= \frac{1}{N}(\frac{E_X^H - E_D^H - \sqrt{(E_X^H - E_D^H)^2 + 4j_+^2}}{2j_+}|X_H\rangle + |D_H\rangle) \\ |\mathcal{D}_V\rangle &= \frac{1}{N}(\frac{E_X^V - E_D^V - \sqrt{(E_X^V - E_D^V)^2 + 4j_-^2}}{2j_-}|X_V\rangle + |D_V\rangle) \end{aligned}$$

with N normalising the states to unit length.

Details on simulation

As mentioned in the main text the driving fields acting on the quantum dot are treated as classical laser fields of Gaussian shape given by

$$\begin{aligned}\Omega(\Delta\mathrm{t}, \omega_{\mathcal{P}}, \Theta, \alpha, \mathbf{e_L}) =& \frac{\mathbf{e_L}\Theta}{\sqrt{2\pi\tau_0\tau}} \exp(-\frac{(t-\Delta\mathrm{t})^2}{2\tau^2}) \\ & \exp(-i(\omega_{\mathcal{P}} + \frac{a(t-\Delta\mathrm{t})}{2})(t-\Delta\mathrm{t}))\end{aligned} \tag{S1}$$

with t being the time and Δt the time of arrival, Θ being the pulse area, $\omega_{\mathcal{P}}$ the central frequency of the laser field and

$$\begin{aligned}\tau =& \sqrt{\frac{\mathrm{GDD}^2}{\tau_0^2} + \tau_0^2}, \\ a =& \frac{\mathrm{GDD}}{\mathrm{GDD}^2 + \tau_0^4}\end{aligned} \tag{S2}$$

incorporating the effect of spectral chirp GDD onto the pulse shape in time with a transform limited duration of τ_0.

The vector $\mathbf{e_L}$ describes the orientation of linear polarisation of the laser pulse and with respect to the polarisation basis of the quantum dot ($\mathbf{e_H}$ and $\mathbf{e_V}$) is given as

$$\mathbf{e_L} = \cos\zeta^{\mathcal{P}}\mathbf{e_H} + \sin\zeta^{\mathcal{P}}\mathbf{e_V}. \tag{S3}$$

Here $\zeta^{\mathcal{P}}$ denotes the angle with respect to emission from $|\mathcal{X}_\mathrm{H}\rangle$ (horizontal green line in Fig. 1(D)).

A summary of the used simulation parameters is presented in table S1:

Details on state dynamics

In this section we pay closer attention to the quantum dot state dynamics during the storage and retrieval steps of the protocol, shown in Fig. S1(A) and (B) respectively.

Assuming that the chirped pulse $\mathcal{P}^\mathrm{s}$ induces a nearly adiabatic system evolution along dressed states, see Fig. 3(B), the simulation suggests that the initial $|\mathrm{XX}\rangle$ component (yellow) is transferred first to $|\mathrm{X_H}\rangle$ (light green segment in the middle) and eventually to the dark state $|\mathrm{D_H}\rangle$ (dark green)

while the smaller initial $|X_H\rangle$ component should end up in $|XX\rangle$. This is reflected in Fig. S1(A). The dynamic is dominated by the lowest dressed state which initially corresponds to a large $|XX\rangle$ component. The adiabatic evolution then continuously decreases the $|XX\rangle$ population. The intermediary transfer to $|X_H\rangle$ is clearly seen as an intermediate maximum in the $|\mathcal{X}_H\rangle$ population. The transfer of the initial $|X_H\rangle$ population to $|XX\rangle$ has little impact since it was rather small at the start but is likely responsible for the finding that the $|XX\rangle$ population stays above that of $|\mathcal{X}_H\rangle$.

Phonons induce transitions between the dressed states. The dotted arrow in Fig. 3(B) illustrates a phonon emission that slightly reduces the population of the upper dressed state, which at long times approaches $|XX\rangle$. Thus, we expect a reduction of the $|XX\rangle$ population which is indeed seen in Fig. S1(A) where dotted lines stem from simulations without phonons while solid lines are calculated including phonons. Interestingly, the dark state population after the pulse $\mathcal{P}^s$ is reduced by phonons although the dark state is the lowest dressed state at long times. We attribute this on the one hand to a phonon induced population exchange between the lower two dressed states during the pulse where these states are rather close in energy such that deviations from the adiabatic picture are likely and on the other hand to phonon absorption processes that can lead to a redistribution from a lower to a higher dressed state in particular when theses states are close in energy. This is in line with the observation that phonons increase the $|\mathcal{X}_H\rangle$ population which would also not be expected if the dynamics were to adiabatically follow the dressed states.

Reading Fig. 3(B) from right to left reveals that a time inverted (positively chirped) pulse $\mathcal{P}^r$ reverts population from $|D_H\rangle$ back into $|XX\rangle$. If applied sufficiently long after $\mathcal{P}^s$ ($\Gamma_{\mathcal{D}}^{-1}$ > storage time $\gg \Gamma_{\mathcal{X}}^{-1} > \Gamma_{XX}^{-1}$) the only occupied state, besides $|G\rangle$, is $|\mathcal{D}_H\rangle$ which leads to an almost perfect population inversion from $|\mathcal{D}_H\rangle \rightarrow |XX\rangle$, limited only by decay during the pulse. This process is shown in Fig. S1(B) and highlights the population retrieval evoked by $\mathcal{P}^r$ which transfers the dark state occupation back into bright states.

Estimation of g-factors

We estimate the combined g-factors ($g_{hx}+g_{ex}$) by stimulating from $|XX\rangle$ into an equal superposition of $|\mathcal{X}_H\rangle$ and $|\mathcal{D}_H\rangle$ and recording time-resolved photon emission. We then extract the decay times of the participating rates by fitting a bi-exponential decay to the data. Repeating this for a set of different magnetic fields and fitting to our numerical model lets us estimate the combined Landé

g-factors (see Fig. S2):

$$(g_{hx} + g_{ex}) = 0.411(3)$$

Together with the observation $(g_{hx} + g_{ex}) \gg (g_{hx} - g_{ex})$ we estimate the individual g-factors as:

$$g_{hx} = g_{ex} \approx 0.205.$$

Experimental setup

A detailed schematic of the experimental setup is presented in Fig. S3.

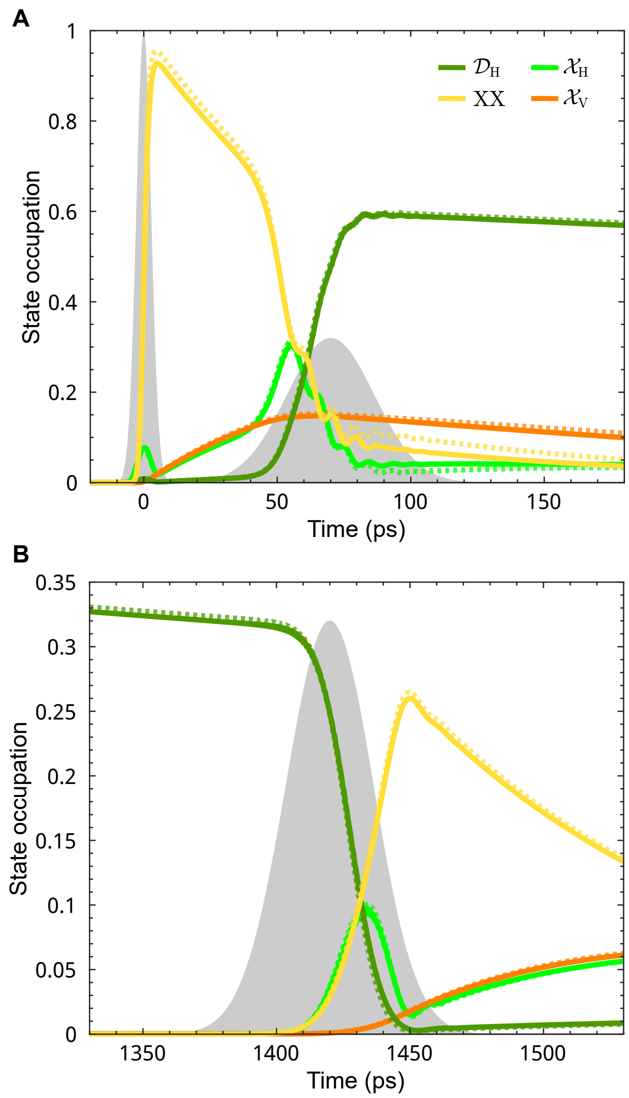

Figure S1: **State dynamics during storage and retrieval**. (**A**) State dynamics during the initialisation pulse $\mathcal{P}^{\mathrm{i}}$ (at 0 ps) and the storage pulse $\mathcal{P}^{\mathrm{s}}$ (at 70 ps). Solid lines are simulations including phonon interaction while dashed lines exclude these interactions. (**B**) Same as in (A) but for the retrieval pulse $\mathcal{P}^{\mathrm{r}}$ at 1420 ps.

Table S1: Simulation parameters. Values are either measured or estimated to our best knowledge from an assemble of quantum dots (QD) on the same sample.

Symbol	QD	$\mathcal{P}^{\mathrm{i}}$	$\mathcal{P}^{\mathrm{s}}$	$\mathcal{P}^{\mathrm{r}}$
$\hbar\omega_{\mathrm{X}}$	1.5628 eV	-	-	-
$\hbar\omega_{\mathrm{D}}$	1.5627 eV	-	-	-
E_{B}	3.6 meV	-	-	-
δ_{X}	11.14 µeV	-	-	-
δ_{D}	11.14 µeV	-	-	-
$(g_{hx} + g_{ex})$	0.41	-	-	-
$(g_{hx} - g_{ex})$	0	-	-	-
Γ_{X}^{-1}	180 ps	-	-	-
$\Gamma_{\mathrm{XX}}^{-1}$	120 ps	-	-	-
QD size	5 nm	-	-	-
T	1.5 K	-	-	-
$\mathrm{B_x}$	3.4 T	-	-	-
$\hbar\omega_{\mathcal{P}}$	-	1.5610 eV	1.5590 eV	1.5590 eV
τ_0	-	2.9 ps	2.9 ps	2.9 ps
GDD	-	0 ps^2	-45 ps^2	+45 ps^2
$\zeta^{\mathcal{P}}$	-	0 rad	0 rad	0 rad
Θ	-	4.5 π	3.5 π	3.5 π
Δt	-	0 ns	0.07 ns	1.42 ns

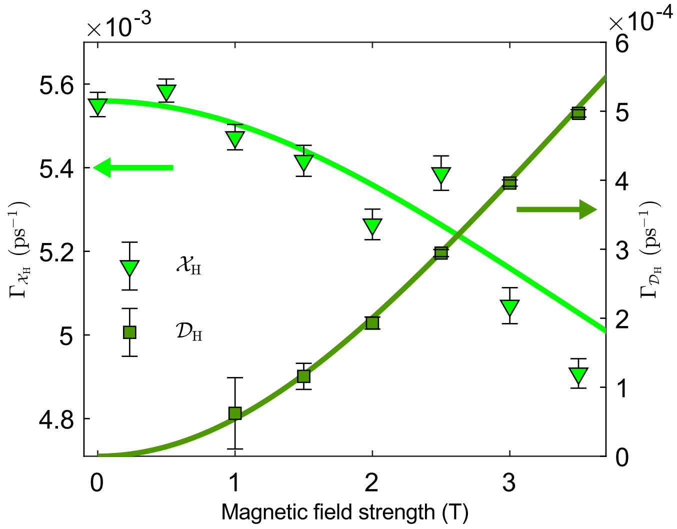

Figure S2: **Decay rate dependence**. Magnetic field dependence of decay rates from $|\mathcal{X}_\mathrm{H}\rangle$ and $|\mathcal{D}_\mathrm{H}\rangle$. Solid lines are best fits to our numerical model resulting in a combined Landé g-factor $(g_{hx} + g_{ex}) = 0.411(3)$.

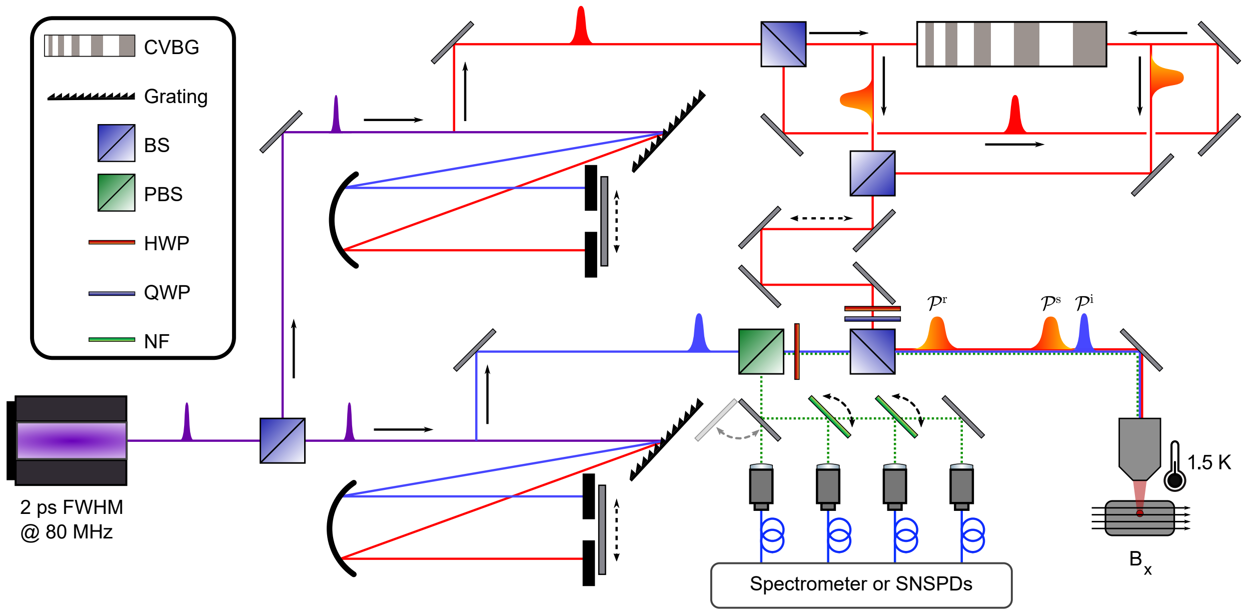

Figure S3: **Schematic of the experimental setup**. For the pulse preparation a pulsed Ti:Sa laser with a pulse duration of ≈ 2 ps FWHM, a repetion rate of ≈ 80 MHz and a mean wavelength at 795 nm is split via a beam-splitter (BS) into two paths to spectrally shape pulses $\mathcal{P}^{\mathrm{i}}$, $\mathcal{P}^{\mathrm{s}}$ and $\mathcal{P}^{\mathrm{r}}$. Using folded 4f pulse shapers consisting of a grating, a concave mirror as well as an motorized slit for spectral tunability. After shaping, laser light is split again and guided into a Mach-Zehnder like setup, leading to a chirped volume Bragg grating (CVBG). The reflected pulses are picking up a spectral chirp of GDD = ± 45 ps^2 depending on the direction of reflection. The path of the 45 ps^2 pulse ($\mathcal{P}^{\mathrm{r}}$) is designed to delay the pulse by ≈ 1.3 ns with respect to the negatively chirped pulse ($\mathcal{P}^{\mathrm{s}}$). In each arm variable neutral density-filters are used for individual power control (not shown). After recombination, an additional variable time delay is introduces to adjust the relative arrival time between $\mathcal{P}^{\mathrm{i}}$ and $\mathcal{P}^{\mathrm{s}}$. Laser pulses are then coupled into the QD experiment setup, employing a cross-polarisation setup consisting of a polarising beam-splitter (PBS), half-wave plates (HWP) as well as quater-wave plates (QWP) for additional polarisation control ($\zeta^{\mathcal{P}}$) and collection basis (ζ^{C}) selection. All pulses are recombined on another BS and directed into the cryostat with a base temperature of 1.5 K in which our quantum dot is placed. The quantum dot is under the influence of a in-plane magnetic field B_x. Finally, the emitted photons from the quantum dot are coupled into a monochromator, consisting of reflective notch-filters (NF) on a rotation mount for frequency selection and pump light suppression, leading to either superconducting nano-wire single photon detectors (SNSPDs) or a spectrometer.
